# Benzene Exposure and Lung Cancer Risk: A Systematic Review and Meta-Analysis of Human Studies

**DOI:** 10.3390/ijerph21020205

**Published:** 2024-02-09

**Authors:** Manuela Chiavarini, Patrizia Rosignoli, Beatrice Sorbara, Irene Giacchetta, Roberto Fabiani

**Affiliations:** 1 Department of Biomedical Sciences and Public Health, Section of Hygiene, Preventive Medicine and Public Health, Polytechnic University of the Marche Region, 60126 Ancona, Italy; m.chiavarini@staff.univpm.it; 2 Department of Chemistry, Biology and Biotechnology, University of Perugia, 06123 Perugia, Italy; patrizia.rosignoli@unipg.it (P.R.); b.sorbara01@gmail.com (B.S.); 3 Department of Medicine and Surgery, Section of Public Heath, School of Hygiene and Preventive Medicine, University of Perugia, 06123 Perugia, Italy; irene.giacchetta@auslromagna.it

**Keywords:** benzene, lung cancer, mortality, incidence, occupational exposure, meta-analysis

## Abstract

Lung cancer is a leading cause of death with nearly 1.8 million deaths estimated worldwide in 2020. Although benzene is classified as a human carcinogen (Group 1) on the basis of its association with acute myeloid/non-lymphocytic leukaemia, there is still limited evidence that it may influence lung cancer risk. This study examined the potential link between benzene exposure and risk of lung cancer using a systematic review of epidemiological studies and meta-analysis. We searched through PubMed, Web of Science and Scopus databases up to 10 February 2023 to identify all articles on the association between benzene exposure and lung cancer (incidence or prevalence) and/or mortality. We extracted the risk estimates of the highest and the lowest reported categories of benzene exposure and conducted a meta-analysis using a random-effects model. Heterogeneity and publication bias were analysed using an I^2^ test and funnel plots asymmetry, respectively. Twenty-one studies were included in the final analysis, with a total of 10,750 lung cancer cases and 2899 lung cancer deaths. Overall, risk estimates of lung cancer prevalence and mortality in association with benzene exposure were 1.20 (*n* = 14; 95% CI 1.05–1.37) and 1.15 (*n* = 13; 95% CI 1.02–1.30), respectively. In all cases, heterogeneity was quite large, while no significant publication bias was observed. When only studies that adjusted for smoking habit were selected, the risk for lung cancer increased by up to 34% (*n* = 9; 95% CI 1.10–1.64). Our data, which show a strong association between benzene exposure and lung cancer risk, may have important public health implications. However, further studies are needed to identify the lung cancer risk associated with benzene exposure considering different smoking conditions.

## 1. Introduction

Lung cancer is the second most commonly diagnosed cancer (more than 2.2 million new cases diagnosed in 2020) and remains the leading cause of cancer death with nearly 1.8 million deaths estimated worldwide in 2020 (18.0% of the total cancer deaths) [1]. Incidence and mortality rates of lung cancer are roughly twice higher in men than in women, and three to four times higher in transitioned countries than in transitioning countries. These differences are due to the different prevalence of cigarette smoking, which is by far the strongest risk factor for lung cancer. Indeed, in the Western world more than 80% of lung cancer cases are attributable to smoking. However, this disease is multifactorial and other determinants may significantly influence its incidence rate [2].

Occupational exposures and air pollution in living environments contribute to the burden of lung cancer with an attributable fraction estimated in the range 5–20% [3]. Indeed, the International Agency for Research on Cancer (IARC) classified both outdoor air pollution and particulate matter (PM) as human carcinogens (Group 1) on the base of sufficient epidemiological evidences derived especially from lung cancer [4]. Further supporting this evidence, a recent meta-analysis reported a significant 14% and 7% increment of lung cancer mortality in association with PM_10_ and PM_2.5_ exposure, respectively [5]. Similarly, a significant positive association between long-term outdoor nitrogen dioxide (NO_2_) exposure and lung cancer mortality in cohort studies was observed with an increment of risk of 8% [6].

In addition to PM and NO_2_, polluted air contains other gas components such as volatile organic compounds (VOCs) which may deeply influence human health. Among different compounds classified as VOCs, benzene is certainly the most studied in relation to its potential human adverse effects [7]. Benzene sources in the air can be both natural and anthropogenic. In the first case, sources include emission from forest fires and volcanoes. Anthropogenic emission of benzene include the incomplete combustion of fossil fuels such as crude oil and gasoline, in industrial processes and motor vehicles [8]. In addition, benzene is widely used in chemical industries both as a solvent and as a reactant due to its primary importance for the synthesis of many compounds such as plastics, resins, and other fibres. Therefore, the large global demand for benzene stood at 52.94 million tonnes in 2020, and it is expected to reach 76.04 million tonnes by 2030 [9].

Benzene remains in the vapor phase in the air. The lifetime of benzene in air ranges from a few hours to days and is dependent on the environmental conditions and the presence of other pollutants. The most important mode of degradation of benzene in the environment is through oxidation by hydroxyl radicle and its subsequent removal by rain [8]. In spite of several efforts to control benzene pollution, it remains one of the most dangerous contaminants in urban air. The maximum desirable value under the Directive 2008/50/EC on air quality in Europe is 5 µg/m^3^ which refers to the annual average concentration in urban outdoor areas [8]. Due to its high volatility, human exposure to benzene mainly occurs via inhalation. Benzene also penetrates skin, but the degree of dermal absorption is rather low. Consequently, the ubiquity of benzene in the environment makes human exposure widespread and unavoidable.

As early as 2012, IARC classified benzene as carcinogenic to humans (Group 1) on the basis of its association with acute myeloid/non-lymphocytic leukaemia [10]. Accordingly, several meta-analyses have recently documented the association between benzene exposure and different haematological malignancies [11,12]. A further 2018 IARC monograph has confirmed the classification of benzene on Group 1 on the basis of sufficient evidence demonstrating its carcinogenicity toward different organs of experimental animals [13]. However, the working group reported that there is still “limited evidence that benzene causes lung cancer” [13].

Given that several epidemiological studies have investigated the potential effect of benzene on the onset of lung cancer with contrasting results, the aim of this study was to rigorously examine the literature available reporting an association between benzene exposure and human lung cancer risk. To this end, all studies reporting both prevalence and mortality risk for lung cancer in association with benzene exposure have been considered. We understand that this paper is the first to examine the effect of benzene exposure on lung cancer risk through a systematic review and meta-analysis.

## 2. Materials and Methods

In this study, the standard procedures for conducting and reporting a meta-analysis according to the COSMOS-E (Conducting Systematic Reviews and Meta-Analyses of Observational Studies of Etiology) statement were followed [14]. The study protocol was registered in the International Prospective Register of Systematic Reviews (www.crd.york.ac.uk/PROSPERO/, registration No: CRD42023398500) (accessed on 21 February 2023).

We defined the PECO (Population, Exposure, Comparator, Outcome) questions as follows: participants were the general population or populations exposed to benzene; exposure was the exposure concentration of benzene; comparison subjects were those with the lowest benzene exposure; outcomes were lung cancer incidence or mortality [15]. In other words, is the higher dose compared with the lower dose of benzene exposure associated with lung cancer incidence or mortality?

### 2.1. Search Strategy and Data Source

A comprehensive literature search, without restrictions, was carried out until 10 February 2023 using PubMed (http://www.ncbi.nlm.nih.gov/pubmed/) (accessed on 10 February 2023), Web of Science (http://wokinfo.com/) (accessed on 10 February 2023), and Scopus (https://www.scopus.com/) (accessed on 10 February 2023) databases to identify all original articles on the association between benzene exposure and lung cancer (oncidence or prevalence) and/or mortality. The following key words were used: (cancer OR tumour OR neoplasm OR “neoplastic disease” OR mortality) AND benzene AND lung. In addition, to identify additional relevant publications, we manually examined the reference lists of the included articles and recent relevant reviews.

### 2.2. Selection Criteria

Of the selected articles, only those that met the following criteria were included: (i) cohort, case-control, and cross-sectional or descriptive (ecological) study design; (ii) evaluated the association between benzene exposure and risk of lung cancer (incidence or prevalence) and mortality; and (iii) presented odds ratio (OR), relative risk (RR), hazard ratio (HR), incidence rate ratios (IRR), and standardized incidence/mortality ratio (SIR/SMR) estimates with 95% confidence intervals (CI). Studies that reported lung cancer risk in occupationally exposed subjects without identifying those specifically exposed to benzene were excluded. Independently, two authors evaluated titles and abstracts, and the studies meeting the inclusion criteria were selected for further full-text analyses. A third author helped to resolve any discrepancies after careful discussion. The publication with the highest number of cases was selected when there were several publications of the same study. The list of selected studies, the removal of duplicates, and the selection of studies of interest were managed with Zotero.

### 2.3. Data Extraction and Management

From each selected study, we extracted the following information: the first author’s last name, year of publication, and country; the study design and name (if any); sample size (incident/prevalent cases, number of cases/controls/death); age; duration of follow-up for cohort studies; exposure assessment method; types of lung cancer; benzene dose comparisons; risk estimated (OR, RR, HR, IRR, SIR, SMR) with 95% confidence intervals; p for trend (in the case of dose-response data); and variables used to adjust the risk value. If more than one risk value was reported, we selected the one obtained by taking into account the greatest number of confounding factors (full-adjusted model).

### 2.4. Assessment of the Level of Evidence

To assess the quality of the evidence, the GRADE methodology for environmental and occupational health studies was used [15,16]. For each study, the risk of bias was defined using a modified version of ROBINS-I as reported previously [17,18]. The following seven aspects were considered: confounding factors, participant selection, exposure classification, deviation from intended exposures (change of exposure levels over time), missing data, measurement of outcomes, and the reported results selection [17,18]. At the end, we classified the quality of evidence as follows: high, moderate, low, or very low.

### 2.5. Statistical Analysis

The overall effect-size statistic estimated was carried out considering the risk of lung cancer associated with the highest versus the lowest level of benzene exposure. Due to the different techniques used in various studies to express exposure to benzene, it was not possible to standardize the exposure parameters prior to conducting the meta-analysis. In the case of the study that assessed benzene exposure using urinary biomarkers, similarly to the other cases, we extracted the risk values for the highest quartile of exposure. The risk values of the multivariable models were selected by considering those that took into account the greatest number of potential confounding variables. Due to the high heterogeneity, a random-effects model and inverse variance weighting methods were used to calculate the sum of RR/OR and the 95% confidence intervals. An effect was considered statistically significant when a two-tailed *p* < 0.05 was obtained. The heterogeneity across studies was evaluated using both a chi-square-based Cochrane’s Q statistic and the I^2^ statistic [19]. The I^2^ value can range from 0 to 25% (indicating no heterogeneity), from 25 to 50% (indicating moderate heterogeneity), from 50 to 75% (indicating large heterogeneity), and from 75 to 100% (indicating extreme heterogeneity) [20].

Since the publication probability depends on the results obtained in the study, the data of the meta-analysis can be influences by so-called “publication bias”. To quantify this publication bias, we used the methods previously reported by Begg and Mazumdar [21] and by Egger et al. [22]. The graphical observation of the funnel plot asymmetry is at the core of both methods. In the first case, the method considers the rank correlation between the effect estimates and their sampling variances [21]. In the second case, the method considers the intercept from the regression of standard normal deviates against precision. The funnel plot was considered asymmetric when the intercept of Egger’s regression line deviated from zero, with *p* values <0.05. When a potential bias was highlighted, a further sensitivity analysis was conducted to assess the robustness of the combined effect estimates.

A sensitivity analysis was also performed to highlight the influence of a single study on the overall risk estimate. One single study at a time was omitted; the results obtained were reported. All analyses were carried out using the statistical program ProMeta version 3.0 available online from Internovi (Cesena, Italy).

## 3. Results

### 3.1. Study Selection

**Figure 1:**
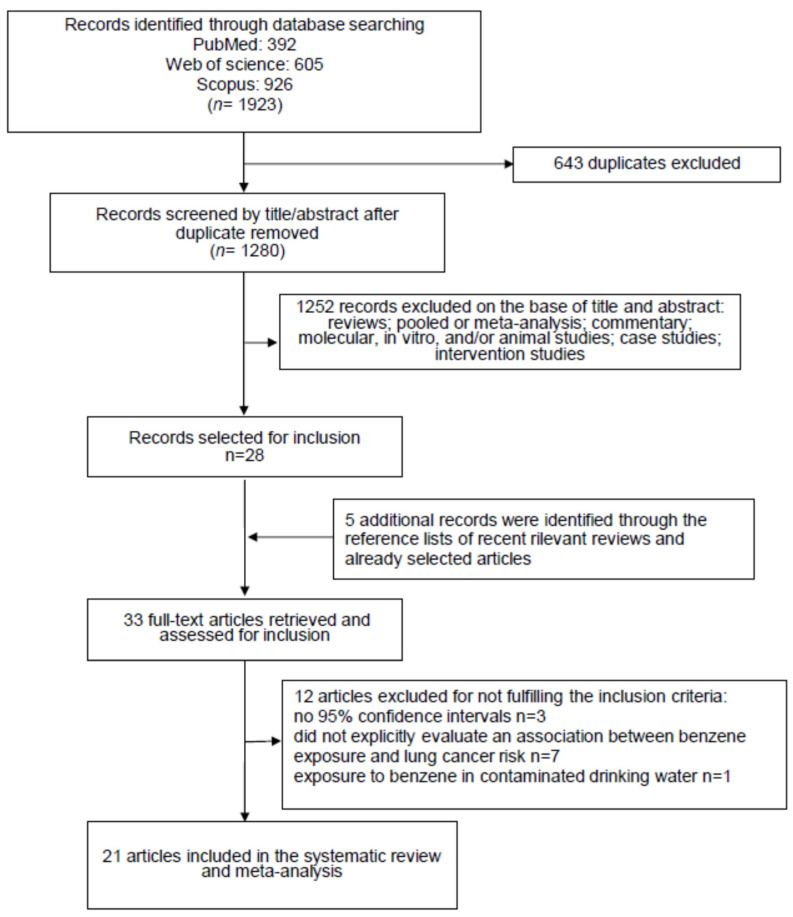
Flow diagram of the systematic literature search on benzene exposure patterns and lung cancer risk.

The primary literature search, carried out on three different databases (PubMed: *n* = 392, Web of Science: *n* = 605 and Scopus: *n* = 926) produced a total of 1923 items. After the removal of 643 duplicates, 1280 records were selected for revision on the basis of their title and abstract (Figure 1).

This procedure led us to exclude 1252 articles because they were not epidemiological studies reporting data regarding the effect of benzene exposure on lung cancer risk. The remaining twenty-eight items were read in full. From the analysis, the reference lists of these articles, and considering also the relevant reviews recently published, we identified five interesting publications to include. Afterwards, 12 items did not meet the inclusion criteria and were barred. The exclusion reasons were as follows: three studies did not report the 95% confidence intervals; seven did not report the risk of lung cancer associated with benzene exposure; one study reported that lung cancer death correlated with exposure to benzene, considering only the contaminated drinking water; one study reported duplicated results of another further study. Therefore, at the end of the selection process, 21 studies were selected [23,24,25,26,27,28,29,30,31,32,33,34,35,36,37,38,39,40,41,42,43], and used for the systematic review and meta-analysis (Figure 1).

### 3.2. Study Characteristics

The general characteristics of the 21 studies, published between 1983 and 2022, that evaluated the association between benzene exposure and lung cancer risk were extracted from the original articles (Table 1).

Eleven studies considered lung cancer incidence or prevalence [23,24,25,26,27,30,32,34,36,37,39], eight studies reported lung cancer mortality [28,29,33,38,40,41,42,43], and three studies reported both incidence or prevalence and mortality [31,35,37]. The indices used to estimate lung cancer risk associated with benzene exposure were OR in seven studies [25,27,30,32,34,36,39], RR in four studies [28,33,40,41]), HR in one study [23]), IRR in two studies [24,26]), and SIR/SMR in seven studies [29,31,35,37,38,42,43]. Overall, 10,750 lung cancer cases and 2899 lung cancer deaths were considered.

Very importantly, of the 21 selected studies, only 10 considered cigarette smoking as a variable to adjust lung cancer risk values [23,24,25,27,30,32,33,34,36,39].

Regarding benzene exposure, fourteen studies evaluated occupational exposure [25,27,28,29,31,35,36,37,38,39,40,41,42,43], five evaluated residential exposure [23,24,26,32,33], and two evaluated general exposure using a biomarker [30,34]. In particular, in eight studies the concentration of benzene exposure was not reported; the subjects were simply classified as unexposed and exposed [25,28,31,35,36,40,41,42]. In three studies the benzene exposure was expressed as ppm [37,38,43], while in four studies the benzene exposure was reported as an increment expressed either as ppb or µg/m^3^ [23,24,32,33]. Only one study reported the cumulative benzene exposure [29], whereas the remaining five studies reported a dose-dependent lung cancer risk according to the exposure intensity and dividing the population in tertiles [26], quartiles [30,34], and different levels of exposure (low, medium, and high) [27,39].

Of the 21 selected studies, only 2 reported risk values stratified by the histologic type of lung cancer [25,39]. Ten studies were conducted on men [25,27,30,31,34,35,39,41,42,43], eight on men and women together [23,24,29,32,33,36,37,38], and three studies were conducted on men and women separately [26,28,40].

Regarding the study designs, six were case-control studies, of which five considered lung cancer disease [25,27,32,36,39] and one considered mortality [41]. In addition, two were “nested” case-control studies that used the concentration of S-phenylmercaptic acid in urine as biomarker to estimate benzene exposure. Of these, the first study was conducted for non-smokers [30], while the second study was conducted for smokers [34]. All the other 13 studies can be grouped as “cohort studies”; although, they were conducted with different methodological approaches, as shown below. Six were retrospective cohort studies [28,29,31,35,38,43], all looking at lung cancer mortality with a total of 884 deaths. Four were cohort studies, two on lung cancer mortality [33,40], one on incidence [23], and one on both incidence and mortality [37]. The two descriptive studies were “spatial” and considered the lung cancer prevalence in the entire population of Tehran (Iran) [24] and Monfalcone with 13 surrounding municipalities (Italy) [26], respectively. In both studies, lung cancer risk was expressed as IRR based on 2,729 lung cancer cases. One study was defined as a historical prospective [42].

Finally, regarding the region, seven studies were conducted in the United States [23,29,36,38,41,42,43], four in Canada [25,32,33,39] and China [28,30,34,40], two in Korea [31,35], and one each in Iran [24], Italy [26], France [27], and the United Kingdom [37].

### 3.3. Risk of Bias

In general, most studies considered for inclusion in the meta-analysis may be at risk of bias. In our case, 9 studies had a risk of bias that was considered “serious”, and for the 12 remaining studies, the risk of bias was “critical” (Supplementary Materials, Table S1). The serious or critical risk of bias registered for the studies included in our meta-analysis was primarily due to the use of an inappropriate analysis method that controlled for important confounding variables, which in many cases were not appropriately measured.

### 3.4. Meta-Analysis

**Figure 2:**
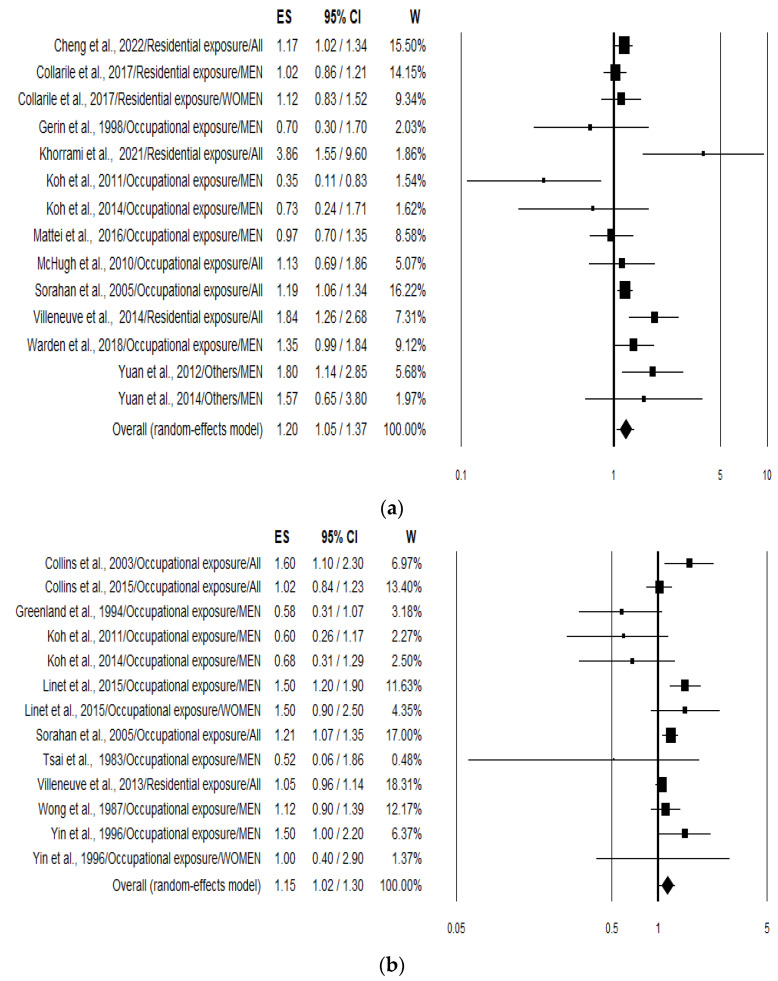
Forest plots showing the lung cancer risk expressed considering incidence and prevalence (**a**) [23,24,25,26,27,30,31,32,34,35,36,37,39] and mortality; (**b**) [28,29,31,33,35,37,38,40,41,42,43] associated with the highest benzene exposure compared with the lowest benzene exposure.

The overall analysis (pooling incidence and mortality risk values) of the 21 studies pooled together (*n* = 27) yielded a combined risk estimate for lung cancer in association to benzene exposure of 1.17 (95% CI 1.08–1.28; *p* < 0.001). Analysing data separately resulted in an increased risk of 20% (1.20; 95% CI 1.05–1.37; *p* = 0.007) (Figure 2a) and 15% (1.15; 95% CI 1.02–1.30; *p* = 0.023) (Figure 2b) for lung cancer incidence or prevalence and mortality, respectively. Heterogeneity was quite large with an I^2^ of 54.19 and 56.55 for incidence and mortality, respectively (Table 2).

Additional results of the stratified analysis were performed according to smoking status (considered as a confounding variable and used to adjust cancer risk values), type of benzene exposure, sex, study design, and region (Table 2). When smoking status was considered, the risk increased to 26% (1.26; 95% CI 1.08–1.48; *p* = 0.005) and 34% (1.34; 95% CI 1.10–1.64; *p* = 0.005) for all studies and for incidence studies, respectively. Heterogeneity was evident in both cases (I^2^ 62.81 and 55.31). Stratifying the analysis according to the type of benzene exposure (Residential/Occupational) and the increment of lung cancer risk resulted in being statistically significant in both cases (Table 2). Furthermore, when S-phenylmercaptic acid in urine was used as biomarker to estimate benzene exposure (only two studies), a statistically significant 75% increased risk was observed (*p* = 0.007). On the other hand, stratification based on sex produced a significant increment of the lung cancer risk only in the group where males and females were considered together (Table 2).

Regarding the study design, pooling together the eight case-control studies, with a total of 4919 cases and 6336 controls, the increment of lung cancer risk (+21%) resulted in being not statistically significant when considering all studies, while becoming significant (+32%) when considering only the incidence and prevalence studies (1.32; 95% CI 1.05–1.66; *p* = 0.019) (Table 2). In this case, heterogeneity was moderate (I^2^ = 44.02%, *p* = 0.097). Conversely, in the case of cohort studies the increment of lung cancer risk was statistically significant when considering all studies (1.15; 95% CI 1.06–1.26; *p* = 0.001) and mortality studies (1.17; 95% CI 1.05–1.32; *p* = 0.006), but was not significant when considering the incidence and prevalence studies (1.12; 95% CI 0.96–1.32; *p* = 0.147) (Table 2). Again, heterogeneity was quite high.

Finally, stratifying according to the continent where the study was conducted, statistically significant incidence results were obtained in both North America and Europe, but not in Asia (Table 2).

### 3.5. Publication Bias

**Figure 3:**
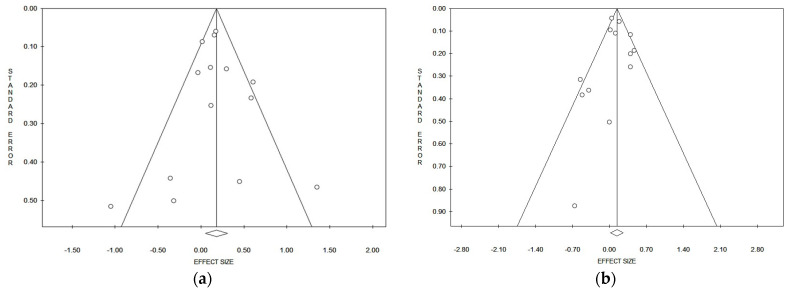
Funnel plots of the meta-analysis on lung cancer incidence/prevalence (**a**) and mortality (**b**) risk.

Considering the pooled data, and applying both the Egger and Begg tests (Table 2), and funnel plots asymmetry, no evident publication bias could be detected for lung cancer risk referred to in the incidence/prevalence studies (Figure 3a) and mortality studies (Figure 3b). In the strata analysis, the Egger test suggested a significant publication bias in the case of all studies that referred to males and females together (*p* = 0.029), while the Begg test gave a positive result on mortality in Asia (*p* = 0.039) (Table 2). No other significant publication biases were observed (Table 2).

### 3.6. Sensitivity Analysis

Investigation into the effect that a single study can have on the value of lung cancer risk suggested that estimates were in some cases changed by a single study. The removal of Greenland et al. 1994 [41] and Koh et al.’s 2011 [35] studies increased the risk values, which in some cases become statistically significant (Supplementary Material, Table S2). In particular, the removal of Greenland et al. 1994 [41] caused the increment of pooled risk in the case–control subgroup (1.32; 95% CI 1.05–1.66; *p* = 0.019), while the removal of Koh et al. 2011 [35] caused the increment of incidence risk in the cohort subgroup (1.15; 95% CI 1.01–1.31; *p* = 0.037) (Supplementary Table S2). Conversely, the removal of studies by Warden et al. 2018, Villenueve et al. 2014, and Sorahan et al. 2005 [25,32,37] caused a decrease of lung cancer risk which becomes in some cases not statistically significant (Supplementary Material, Table S2).

## 4. Discussion

To the best of our knowledge, this is the first systematic review and meta-analysis to summarize the relationships between benzene exposure and lung cancer risk. We considered data regarding both lung cancer incidence/prevalence and mortality, and analysed them both together and separately. We found a statistically significant association between benzene exposure and lung cancer risk in studies both considering the incidence/prevalence and mortality. In this last case, stratification based on the study design (exclusion of the single case–control study) resulted in an increment of mortality risk for lung cancer in the cohort studies. It should be considered, however, that these conclusions are based on a “low” level of evidence as shown using the GRADE approach.

Although more than 80% of all new cases are attributable to smoking, lung cancer is a multifactorial disease, which may be influenced by other important determinants. The increment in recent years of lung cancer incidence in never smokers indicates that other important risk factors need to be investigate [44]. Accordingly, several recent meta-analyses have suggested that different air pollutants, in particular PM_10_/PM_2.5_ and NO_2_, also contribute significantly to the increment of lung cancer incidence and mortality [5,6]. Even more important to consider is the deleterious effects that occupational exposure (gases/fumes, vapours, dusts, fumes, and aromatic solvents) may have on the decline of lung function and cancer [45]. Our meta-analysis further support this evidence, showing that exposure to benzene, an important compound belonging to the VOCs class and present both in the polluted urban/industrial air and in the working place, is associated with a statistically significant 20% increment of lung cancer risk (expressed as incidence rate). Our data also confirm and support the latest IARC classification, which included benzene in Group 1 as a human carcinogen, based primarily on its hematotoxic and leukemogenic properties [13]. The important role played by smoking on lung cancer incidence is also evident from the data reported in the present study. In fact, the stratified analysis yielded a statistically significant 34% increase in lung cancer incidence in association with benzene exposure only when considering studies adjusted for smoking, whereas no effect was evident when considering studies not adjusted for smoking (Table 2). However, in the analysis of lung cancer mortality, based mainly on data unadjusted for smoking habits, we still observed a statistically significant increase in risk (Table 2). Unfortunately, most of the mortality studies (11 of 12) did not contain information on smoking habits, making the results not particularly reliable in this context.

As the above reported, more incisive are our results on lung cancer incidence/prevalence. In this case, the stratified analysis according to the type of exposure (residential vs. occupational) showed a statistically significant 28% increase of risk in association with residential benzene exposure, while a smaller (8%) and not statistically significant effect was observed for occupational exposure. This result may be surprising because occupational benzene exposure concentrations for workers should be higher in respect to those of residents in polluted areas [46]. One possible explanation of this phenomenon could be related to the so called “Healthy worker” effect (HWE) which is a special type of selection bias, typically seen in observational studies of occupational exposures with an improper choice of comparison group (usually the general population) [47]. For instance, when SMRs are calculated using the general population as a reference, the values may be underestimated due to the mortality of the occupational population. In any case, previous findings have clearly indicated that benzene exposure in the workplace was associated with increased risk of several neoplasms, including leukemia [48,49], multiple myeloma [50], and non-Hodgkin lymphoma [51]. Workplace exposure is often complex and related to other important carcinogens, such as other VOCs, polycyclic aromatic hydrocarbons, and heavy metals. Therefore, for an accurate risk assessment, these variables should always be taken into account to adjust the results. Instead, only a few studies included in this meta-analysis considered exposure to other occupational carcinogens [25,39] as matched or adjusted variables. Furthermore, our data may be difficult to explain due to the low number of studies included in the analysis. Indeed, the sensitivity analysis showed evident variation by omitting single studies. In particular, removal of the study by Koh et al., 2011, [35] resulted in a statistically significant effect on lung cancer risk (1.17; CI 1.05–1.29; *p* = 0.003) associated with workplace benzene exposure.

Another important characteristic that may influence lung cancer risk in association with benzene exposure is sex. Animal studies suggest that males have a greater susceptibility to the adverse health effects of benzene, while human studies indicate that women have a significantly higher risk of blood system effects than men in a similar situation of benzene exposure [52]. Unfortunately, our sex-stratified analysis produced dubious results, as very few studies on women were conducted, and a significant increase in lung cancer incidence was only observed when studies that considered males and females together were pooled.

Regarding the biological plausibility of benzene carcinogenicity in the lungs, this compound has been extensively studied in the past, especially in relation to haematological malignancies due to its well-known myelotoxicity. The haematotoxicity of benzene is mediated by the production of several toxic metabolites and reactive oxygen species capable of causing DNA damage and mutations in target cells [53]. Cytogenetic damage correlated to benzene exposure is well known. Quantitative meta-analysis have reported a clear association between occupational exposure to benzene with chromosomal aberrations (CAs) and micronuclei (MN) as markers of DNA damage [54,55]. Although the metabolism of benzene takes place mainly in the liver, the lung is also capable of metabolising it, making it possible to form toxic metabolites directly in situ [56]. Benzene is mainly emitted in the air, so human exposure occurs mainly by inhalation, making the lung the first target for its toxicity. Recently, a significant positive relationship between impaired lung function and occupational exposure to benzene has been demonstrated [57].

The present systematic review and meta-analysis has several strengths and limitations. Unlike previous meta-analyses on benzene exposure and risk of non-Hodgkin lymphoma [12], leukemia [48], and chronic myeloid leukemia [49], which did not distinguished the incidence of the disease from the mortality for the disease, we were able to analyse incidence/prevalence and mortality separately. In addition, our results showing the increased risk of disease incidence were obtained from a substantial number of 10,750 lung cancer cases spread across three continents. Moreover, although we observed a consistent heterogeneity, probably due to the results from the primary studies included, no evident publication biases were detected.

On the other hand, our results should be interpreted with caution because of several limitations. Due to the relatively small number of studies included, our meta-analysis may restrict the statistical power to evidence an association and may reduce the generalisability of the results. This is more evident in the stratified analysis on the same important parameters including sex and type of benzene exposure, which produced in same cases complicated results. Although it is well known that smoking significantly increases the risk of lung cancer, many studies reported unadjusted data for this important confounding factor. Further studies should focus on this issue and try to identify the possible effects of benzene exposure separately for different smoking conditions (never smoker, current smoker, and former smoker). Another important limitation of the studies that were included is the quality of the benzene-exposure assessment. In some studies, the exposure dose was not reported and in others, exposure estimates were reported as a slope factor (increment) making exposure assessment extremely heterogeneous. Even for studies with good qualitative assessment of exposure, as various solvents are often used simultaneously and sometimes as components of complex mixtures, in many cases, it is difficult to extrapolate the risk associated with individual substances. Nevertheless, there is a possibility that misclassification might have occurred in some studies. In the future, it will be important to adjust the risk values to consider different concomitant exposure and to more accurately identify the “exposure dose”. In addition, the indoor contribution to the benzene exposure could be considered, and further longitudinal studies should adjust for smoking and co-exposure in the statistical analysis.

## 5. Conclusions

In this study, we reviewed the available evidence showing an association between benzene exposure and lung cancer risk. The meta-analysis has shown a significant increment of both incidence and mortality for lung cancer in benzene-exposed subjects. Furthermore, the stratified analysis performed considering only studies that adjusted for smoking habits indicated a marked increase in lung cancer risk in association with benzene exposure. These data suggest that smoking is an important risk factor, which if not taken into account may mask the effect of other less-impactful risk factors. There is, therefore, a need for further studies aimed at identifying the lung cancer risk associated with benzene exposure, considering different smoking conditions (never smoker, current smoker, and former smoker).

## Figures and Tables

**Table 1:** Characteristics of the studies included in the systematic revision and meta-analysis on the association between benzene exposure and lung cancer risk.

First Author, Year Location Reference	Study Design, Name and Population Cases/Controls Incident Cases Dead Age Follow-Up	Assessment of Exposure	Types of Lung Cancer Incidence/ Mortality	Benzene Exposure	OR/RR/HR/IRR ^1^/SMR ^2^/SIR ^3^ (95% CI)	Matched or Adjusted Variables
Cheng et al., 2022 USA [23]	Cohort Multiethnic cohort study (MEC) Population: 97,288 Incident cases: 2796 Age: 45–75 years Follow-up: 17 years	Residential exposure Air-monitoring data	All types Incidence	Exposed 1 ppb increment	Men + Women (HR) 1.17 (1.02–1.34)	Age, race/ethnicity, sex, education, marital status, smoking, family history of lung cancer, occupation, socioeconomic status, nonsteroidal anti-inflammatory drugs, body mass index, alcohol, physical activity, energy intake, meat intake.
Khorrami et al., 2021 Iran [24]	Descriptive (ecologic, follow-up) Population: 9,134,708 Incident cases: 1653 Age: median 65.5 years Follow-up: 3 years	Residential exposure Land use regression models	All types Incidence	Exposed 10 µg/m^3^ increment	Men + Women (IRR) 3.86 (1.55–9.60)	Age, sex, socioeconomic status, life expectancy, smoking prevalence
Warden et al., 2018 Canada [25]	Population based case–control (Male) Cases: 894 Age: 64.2 ± 7.8 years Controls: 733 Age: 65.0 ± 7.6 years	Occupational exposure Interview: lifestyle and job features	All types		Men (OR)	Age, smoking (CSI: cumulative smoking index), respondent status, ethnolinguistic group, years of education, median household income as well as Groups 1 and 2A occupational carcinogens: diesel engine emissions, crystalline silica, coke dust, coal dust, and welding fumes.
			Incidence	Unexposed	1.00 (Ref.)	
				Ever exposed	1.35 (0.99–1.84)	
			Adenocarcinoma	Unexposed	1.00 (Ref.)	
				Ever exposed	1.35 (0.90–2.02)	
			Squamous carcinoma	Unexposed	1.00 (Ref.)	
				Ever exposed	1.36 (0.92–2.00)	
			Small cell carcinoma	Unexposed	1.00 (Ref.)	
				Ever exposed	1.51 (0.88–2.56)	
Collarile et al., 2017 Italy [26]	Descriptive (follow-up) Incident cases: 801 men 275 women All age Follow-up: 15 years	Residential exposure Integrated approach based on punctual observations (local environmental monitoring systems) and numerical simulated fields	All types Incidence	µg/m^3^	Men (IRR)	None
				<1.1	1.00 (Ref.)	
				1.1–1.8	1.03 (0.87–1.22)	
				>1.8	1.02 (0.86–1.21)	
					Women (IRR)	
				<1.1	1.00 (Ref.)	
				1.1–1.8	1.09 (0.81–1.46)	
				>1.8	1.12 (0.83–1.52)	
Mattei et al., 2016 France [27]	Population based case–control (Male) ICARE ^4^ study Cases: 2260 Age: 60 ± 9.1 years Controls: 2780 Age: 58 ± 79.9 years	Occupational exposure Interview: lifestyle behaviours and job features Job-exposure matrices (JEM)	All types Incidence		Men (OR)	Age, exposure to asbestos (CEI: Cumulative Exposure Index), smoking (CSI: Comprehensive Smoking Index)
				Unexposed	1.00 (Ref.)	
				Low	1.29 (0.92–1.80)	
				Medium	1.14 (0.80–1.60)	
				High	0.97 (0.70–1.35)	
Linet et al., 2015 China [28]	Retrospective cohort 73,789 exposed Dead: 351 35,504 unexposed Dead: 119 Follow-up: 28 years Age > 12 y at start of first exposure	Occupational exposure Factory and job title-specific information on use of benzene containing materials	All types Mortality		Men (RR)	Age, sex, attained calendar year
				Unexposed	1.00 (Ref.)	
				Exposed	1.5 (1.2–1.9)	
					Woman (RR)	
				Unexposed	1.00 (Ref.)	
				Exposed	1.5 (0.9–2.5)	
Collins et al., 2015 USA [29]	Retrospective cohort 2266 workers Dead: 146 Follow-up: >30 years	Occupational exposure Job-specific exposures	All types Mortality	Cumulative: ≥25 ppm per year	Men + Women (SMR) 1.05 (0.89–1.24)	None
Yuan et al., 2014 China [30]	Nested case–control Shanghai Cohort Study Cases: 82 Age: 58.1 ± 5.2 years Controls: 83 Age: 58.0 ± 5.4 years Lifelong never smokers	Exposure biomarker (S-phenyl mercapturic acid) in urine	All types Incidence		Men (OR)	Age at baseline, neighbourhood of residence at enrolment, years of sample storage, urinary cotinine level Never smokers
				Quartile 1	1.00	
				Quartile 2	1.03 (0.39–2.69)	
				Quartile 3	1.10 (0.44–2.78)	
				Quartile 4	1.57 (0.65–3.80)	
Koh et al., 2014 Korea [31]	Retrospective cohort 14,698 male Incident cases: 5 Death: 9 Age: 20–72 years Follow-up: 4–6 years	Occupational exposure Workers of refinery/petrochemical complex	All types Incidence Mortality	Exposed	Men (SIR) 0.73 (0.24–1.71) Men (SMR) 0.68 (0.31–1.29)	None
Villeneuve et al., 2014 Canada [32]	Case–control Cases: 445 Age: median 66 years Controls: 948 Hospital: 523, 50 years Population: 425 59 years	Residential exposure Land use regression model Interview: lifestyle behaviours	All types Incidence	Interquartile-Range Increase 0.15 μg/m^3^	Men + Woman (OR) Population controls 1.84 (1.26–2.68)	Age, sex, pack-years of smoking, exposure to second-hand smoke, body mass index, family history of cancer, and neighbourhood measures of unemployment and median family income
Villeneuve et al., 2013 Canada [33]	Cohort “Ontario Tax Cohort study” 29,600 men 29,150 women Dead: 1410 Age: >35 years Follow-up: 22 years	Residential exposure Land use regression model	All types Mortality	Interquartile-Range increase 0.13 μg/m^3^	Men + Women (RR) 1.05 (0.96–1.14)	Age, sex, family income, marital status, census area measures of income, immigration, unemployment, land use regression estimate of NO_2_. Indirect adjustment for smoking and body mass index.
Yuan et al., 2012 China [34]	Nested case–control Shanghai Cohort Study Cases: 343 Age: 69.4 ± 6.3 years Controls: 392 Age: 69.1 ± 6.0 years All Smokers	Exposure biomarkers (S-phenyl mercapturic acid) in urine	All types Incidence		Men (OR)	Age, neighbourhood of residence, and duration of biospecimen storage before laboratory analysis, number of cigarettes smoked per day, and number of years of smoking at baseline
				Quartile 1	1.00	
				Quartile 2	1.46 (0.91–2.35)	
				Quartile 3	1.94 (1.23–3.07)	
				Quartile 4	1.80 (1.14–2.85)	
Koh et al., 2011 Korea [35]	Retrospective cohort 8866 male Incident cases: 8 Dead: 5 Follow-up: Mortality 16 years Incidence 9 years Age: 20–74 y	Occupational exposure Work history in refinery and petrochemical companies	All types Incidence Mortality	Exposed	Men (SIR) 0.60 (0.26–1.17) Men (SMR) 0.35 (0.11–0.83)	None
McHugh et al., 2010 USA [36]	Case–control Mexican-Americans Cases: 38 Age: 63.8 ± 11.0 years Controls: 51 Age: 61.5 ± 12.8 years	Occupational exposure Self-reported responses	All types Incidence		Men + Women (OR)	Age, sex, and smoking
				Unexposed	1.00 (Ref.)	
				Exposed	1.13 (0.69–1.86)	
Sorahan et al., 2005 UK [37]	Cohort 4740/5130 Men 352/384 Women Incident cases: 293 Death: 294 Follow-up: 30 years	Occupational exposure Type of industry	All types Incidence Mortality	Exposed ≥ 25 ppm	Men + Women (IRR) 1.19 (1.06–1.34) Men + Women (SMR) 1.21 (1.07–1.35)	None
Collins et al., 2003 USA [38]	Retrospective cohort 4417 workers Dead: 252 Follow-up: >30 years	Occupational exposure Job type at the different plants	All types Mortality	>100 ppm per day	Men + Women (SMR) 1.6 (1.1–2.3)	None
Gerin et al., 1998 Canada [39]	Population-based case–control (Men) Cases: 857 Controls: 1349 Age: 35–70 years	Occupational exposure Interview: lifestyle behaviours and job features	All types		Men (OR)	Age, family income, ethnic group, cigarette smoking, respondent status, arsenic, asbestos, chromium VI, nickel, crystalline silica, beryllium, cadmium, polycyclic aromatic hydrocarbons
			Incidence	Unexposed	1.00 (Ref.)	
				Low	1.1 (0.8–1.5)	
				Medium	0.8 (0.5–1.3)	
				High	0.7 (0.3–1.7)	
			Adenocarcinoma	Unexposed	1.00 (Ref.)	
				Low	1.1 (0.6–1.8)	
				Medium/High	0.9 (0.4–1.9)	
			Squamous carcinoma	Unexposed	1.00 (Ref.)	
				Low	1.3 (0.9–1.9)	
				Medium/High	1.2 (0.7–2.1)	
			Small cell carcinoma	Unexposed	1.00 (Ref.)	
				Low	1.10 (0.6–1.9)	
				Medium/High	0.30 (0.1–0.9)	
Yin et al., 1996 China [40]	Cohort Men and woman Exposed: 74,828 38,833 men 35,995 women Follow-up: 10.5 years Unexposed: 35,805 20,795 men 15,010 women Follow-up: 11.7 years Dead: 125	Occupational exposure Type of industry	All types Mortality		Men (RR)	Age
				Unexposed	1.00 (Ref.)	
				Exposed	1.50 (1.0–2.2)	
					Women (RR)	
				Unexposed	1.00 (Ref.)	
				Exposed	1.00 (0.4–2.9)	
Greenland et al., 1994 USA [41]	Case–control Case: 139 lung cancer death Control: 1202 non-cancer death Age: 21–90 years	Occupational exposure Job history records	All types Mortality		Men (RR)	None
				Unexposed	1.00 (Ref.)	
				Exposed	0.58 (0.31–1.07)	
Wong 1987 USA [42]	Historical prospective 7676 Men Dead: 47 Follow-up: 32 years	Occupational exposure Chemical workers	All types Mortality	Exposed	Men (SMR) 1.12 (0.90–1.39)	Age, race, sex, calendar time
Tsai et al., 1983 USA [43]	Retrospective cohort 454 Men Dead: 2 Age: average at entry 33.8 years Follow-up: 13 years	Occupational exposure Workers in benzene processes or operations	All types Mortality	Exposed 1.34 ± 1.39 ppm	Men (SMR) 0.52 (0.06–1.86)	None

^1^ Incidence rate ratios; ^2^ standardized mortality ratios; ^3^ standardized incidence ratio; ^4^ investigation of occupational and environmental causes of respiratory cancers.

**Table 2:** Results of combined lung cancer risk estimates, heterogeneity, and publication bias, stratified according to different variables and associated with the highest benzene exposure compared with the lowest benzene exposure ^1^.

	Combined Risk Estimate		Test of Heterogeneity			Publication Bias	
	Value (95% CI)	*p*	Q	I^2^ %	*p*	*p* (Egger Test)	*p* (Begg Test)
**ALL** (*n* = 27) ^2^	**1.17 (1.08–1.28)**	<0.001	56.79	54.22	0.0004	0.906	0.574
**Smoking**							
Adjusted (*n* = 10)	**1.26 (1.08–1.48)**	0.005	24.02	62.81	0.004	0.077	0.245
Not adjusted (*n* = 17)	**1.13 (1.02–1.26)**	0.021	32.20	50.31	0.009	0.105	0.161
**Exposure**							
Residential (*n* = 6)	**1.19 (1.02–1.39)**	0.030	17.10	70.76	0.004	0.051	0.091
Occupational (*n* = 19)	**1.14 (1.03–1.27)**	0.015	33.16	45.71	0.016	0.052	0.086
Others: biomarkers (*n* = 2)	**1.75 (1.16–2.63)**	0.007	0.07	0.00	0.788	---	---
**Sex**							
Men (*n* = 15)	1.07 (0.90–1.28)	0.428	32.81	57.34	0.003	0.136	0.299
Women (*n* = 3)	1.19 (0.93–1.54)	0.168	1.06	0.00	0.588	0.900	0.602
Men and Women (*n* = 9)	**1.20 (1.08–1.34)**	0.001	22.75	64.84	0.004	0.029	0.144
**Study design**							
Case Control (*n* = 8)	1.21 (0.93–1.57)	0.159	17.03	58.89	0.017	0.498	0.458
Cohort (*n* = 19)	**1.15 (1.06–1.26)**	0.001	38.43	53.16	0.003	0.970	0.506
**Continent**							
North America (*n* = 11)	**1.15 (1.02–1.29)**	0.022	21.18	52.79	0.020	0.772	0.938
Asia (*n* = 11)	1.23 (0.92–1.63)	0.157	24.64	59.42	0.006	0.179	0.073
Europe (*n* = 5)	**1.15 (1.07–1.24)**	<0.001	4.02	0.48	0.403	0.178	0.142
**INCIDENCE** (*n* = 14)	**1.20 (1.05–1.37)**	0.007	28.38	54.19	0.008	0.799	0.784
**Smoking**							
Adjusted (*n* = 9)	**1.34 (1.10–1.64)**	0.005	17.90	55.31	0.022	0.282	0.297
Not adjusted (*n* = 5)	1.06 (0.89–1.27)	0.494	8.00	49.98	0.092	0.096	0.142
**Exposure**							
Residential (*n* = 5)	**1.28 (1.02–1.61)**	0.031	14.66	72.72	0.005	0.114	0.142
Occupational (*n* = 7)	1.08 (0.89–1.30)	0.460	9.80	38.75	0.134	0.067	0.051
Others: biomarkers (*n* = 2)	**1.75 (1.16–2.63)**	0.007	0.07	0.00	0.788	---	---
**Sex**							
Men (*n* = 8)	1.09 (0.86–1.38)	0.492	14.64	52.20	0.041	0.719	0.458
Women (*n* = 1)							
Men and Women (*n* = 5)	**1.32 (1.09–1.60)**	0.005	11.31	64.64	0.023	0.125	0.142
**Study design**							
Case Control (*n* = 7)	**1.32 (1.05–1.66)**	0.019	10.72	44.02	0.097	0.850	0.881
Cohort (*n* = 7)	1.12 (0.96–1.32)	0.147	15.21	60.54	0.019	0.743	0.176
**Continent**							
North America (*n* = 5)	**1.28 (1.04–1.56)**	0.018	6.97	42.64	0.137	0.864	1.000
Asia (*n* = 5)	1.29 (0.64–2.59)	0.481	14.78	72.93	0.005	0.481	0.142
Europe (*n* = 4)	**1.12 (1.02–1.22)**	0.013	2.92	0.00	0.405	0.348	0.497
**MORTALITY** (*n* = 13)	**1.15 (1.02–1.30)**	0.023	27.62	56.55	0.006	0.838	0.542
**Smoking**							
Adjusted (*n* = 1)							
Not adjusted (*n* = 12)	**1.17 (1.01–1.35)**	0.037	23.11	52.41	0.017	0.314	0.273
**Exposure**							
Residential (*n* = 1)							
Occupational (*n* = 12)	**1.17 (1.01–1.35)**	0.037	23.11	52.41	0.017	0.314	0.273
Others: biomarkers (*n* = 0)							
**Sex**							
Men (*n* = 7)	1.03 (0.77–1.37)	0.867	17.14	64.99	0.009	0.079	0.293
Women (*n* = 2)	1.38 (0.87–2.17)	0.167	0.51	0.00	0.476	---	---
Men and Women (*n* = 4)	**1.14 (1.00–1.29)**	0.049	8.24	63.61	0.041	0.420	0.174
**Study design**							
Case Control (*n* = 1)							
Cohort (*n* = 12)	**1.17 (1.05–1.32)**	0.006	23.18	52.55	0.017	0.818	0.583
**Continent**							
North America (*n* = 6)	1.07 (0.93–1.24)	0.343	9.54	47.58	0.089	0.796	0.573
Asia (*n* = 6)	1.21 (0.91–1.61)	0.199	9.62	48.02	0.087	0.073	0.039
Europe (*n* = 1)							

^1^ The risk estimates were calculated using the random-effects model. ^2^ Amount of data used to calculate the risk. In bold are the statistically significant risk values, and highlighted in gray are the related *p* values.

## Data Availability

The data presented in this study are available on request from the corresponding author.

## Supplementary Materials

The following supporting information can be downloaded at https://www.mdpi.com/article/10.3390/ijerph21020205/s1, Table S1: Risk of bias assessment tool for environmental studies (green colour: very low risk of bias; yellow colour: low risk; orange colour: moderate risk; red colour: high risk). Table S2: Results of sensitivity analysis on stratified data.
