# Supplementary material for: Benzene Exposure and Lung Cancer Risk: A Systematic Review and Meta-Analysis of Human Studies
Source: Int J Environ Res Public Health. 2024 Feb 9;21(2):205. doi: 10.3390/ijerph21020205 (PMC10887806; doi:10.3390/ijerph21020205)
Supplement: Supplementary file 1 [file ijerph-21-00205-s001.zip › ijerph-2811120-supplementary.pdf]

**Supplementary Table S1**. Risk of bias assessment tool for environmental studies (green colour: very low risk of bias; yellow colour: low risk; orange colour: moderate risk; red colour: high risk).

| | Confounding | Selection of participants into the study | Classification of exposures | Deviation from intended exposures | Missing data | Measurement of outcomes | Selection of the reported results | Overall bias |
|---|---|---|---|---|---|---|---|---|
| Cheng et al. 2022 [23] | orange | yellow | yellow | NI[1] | green | green | green | orange |
| Khorrami et al. 2021 [24] | orange | yellow | yellow | NI | green | green | green | orange |
| Warden et al. 2018 [25] | orange | yellow | yellow | yellow | green | green | green | orange |
| Collarile et al. 2017 [26] | red | yellow | yellow | NI | green | green | green | red |
| Mattei et al. 2016 [27] | orange | yellow | yellow | yellow | green | green | green | orange |
| Linet et al. 2015 [28] | red | yellow | yellow | NI | green | green | green | red |
| Collins et al. 2015 [29] | red | yellow | yellow | NI | green | green | green | red |
| Yuan et al. 2014 [30] | orange | yellow | yellow | NI | green | green | green | orange |
| Koh et al. 2014 [31] | red | yellow | yellow | NI | green | green | green | red |
| Villeneuve et al. 2014 [32] | orange | yellow | yellow | | green | green | green | orange |
| Villeneuve et al., 2013 [33] | orange | yellow | yellow | NI | NI | yellow | green | red |
| Yuan et al. 2012 [34] | orange | yellow | yellow | NI | green | green | green | orange |
| Koh et al. 2011 [35] | red | yellow | yellow | NI | green | green | green | red |
| McHugh et al. 2010 [36] | red | yellow | red | NI | green | green | green | red |
| Sorahan et al. 2005 [37] | red | yellow | red | NI | yellow | green | green | red |
| Collins et al. 2003 [38] | red | yellow | yellow | NI | green | green | green | red |
| Gerin et al. 1998 [39] | orange | yellow | yellow | NI | green | green | green | orange |
| Yin et al. 1996 [40] | red | yellow | yellow | NI | green | green | green | orange |
| Greenland et al., 1994 [41] | red | yellow | yellow | NI | yellow | green | green | red |
| Wong et al., 1987 [42] | red | yellow | yellow | NI | yellow | green | green | red |
| Tsai et al. 1983 [43] | red | yellow | yellow | NI | yellow | green | green | red |

1: no information

**Supplementary Table S2**. Results of sensitivity analysis of stratified data.

| | Combined risk estimate | | Sensitivity analysis | | | |
|---|---|---|---|---|---|---|
| | **Value (95% CI)** | **p** | **Effect** | **Study removed** | **Value (95% CI)** | **p** |
| **ALL** (n=27)[2] | 1.17 (1.08-1.28) | <0.001 | No | --- | | |
| **Smoking** | | | | | | |
| Adjusted (n=10) | 1.26 (1.08-1.48) | 0.005 | No | --- | | |
| Not adjusted (n=17) | 1.13 (1.02-1.26) | 0.021 | Yes | Sorahan et al., 2005 [37] | 1.09 (0.94-1.27) | 0.268 |
| **Exposure** | | | | | | |
| Residential (n=6) | 1.19 (1.02-1.39) | 0.030 | Yes | Villeneuve et al., 2014 [32] | 1.11 (0.98-1.26) | 0.110 |
| Occupational (n=19) | 1.14 (1.03-1.27) | 0.015 | Yes | Soharan et al., 2005 [37] | 1.09 (0.94-1.27) | 0.264 |
| Others: biomarkers (n=2) | 1.75 (1.16-2.63) | 0.007 | --- | --- | | |
| **Sex** | | | | | | |
| Men (n=15) | 1.07 (0.90-1.28) | 0.428 | No | --- | | |
| Women (n=3) | 1.19 (0.93-1.54) | 0.168 | No | --- | | |
| Men and Women (n=9) | 1.20 (1.08-1.34) | 0.001 | No | --- | | |
| **Study design** | | | | | | |
| Case Control (n=8) | 1.21 (0.93-1.57) | 0.159 | Yes | Greenland et al., 1994 [41] | 1.32 (1.05-1.66) | 0.019 |
| Cohort (n=19) | 1.15 (1.06-1.26) | 0.001 | No | --- | | |
| **Region** | | | | | | |
| North America (n=11) | 1.15 (1.02-1.29) | 0.022 | No | --- | | |
| Asia (n=11) | 1.23 (0.92-1.63) | 0.157 | Yes | Koh et al., 2011 [35] | 1.45 (1.15-1.82) | 0.001 |
| Europe (n=5) | 1.15 (1.07-1.24) | <0.001 | No | --- | | |
| **INCIDENCE** (n=14) | 1.20 (1.05-1.37) | 0.007 | No | --- | | |
| **Smoking** | | | | | | |
| Adjusted (n=9) | 1.34 (1.10-1.64) | 0.005 | No | --- | | |
| Not adjusted (n=5) | 1.06 (0.89-1.27) | 0.494 | Yes | Koh et al., 2011 [35] | 1.13 (1.03-1.24) | 0.010 |
| **Exposure** | | | | | | |
| Residential (n=5) | 1.28 (1.02-1.61) | 0.031 | Yes | Villenueve et al., 2014 [32] | 1.17 (0.95-1.44) | 0.144 |
| Occupational (n=7) | 1.08 (0.89-1.30) | 0.460 | Yes | Koh et al., 2011 [35] | 1.17 (1.05-1.29) | 0.003 |
| Others: biomarkers (n=2) | 1.75 (1.16-2.63) | 0.007 | --- | | | |
| **Sex** | | | | | | |
| Men (n=8) | 1.09 (0.86-1.38) | 0.492 | No | --- | | |
| Women (n=1) | | | --- | | | |
| Men and Women (n=5) | 1.32 (1.09-1.60) | 0.005 | No | --- | | |
| **Study design** | | | | | | |
| Case Control (n=7) | 1.32 (1.05-1.66) | 0.019 | Yes | Warden et al., 2018 [25] | 1.31 (0.97-1.76) | 0.081 |
| Cohort (n=7) | 1.12 (0.96-1.32) | 0.147 | Yes | Koh et al., 2011 [35] | 1.15 (1.01-1.31) | 0.037 |
| **Region** | | | | | | |
| North America (n=5) | 1.28 (1.04-1.56) | 0.018 | Yes | Warden et al., 2018 [25] | 1.25 (0.94-1.67) | 0.125 |
| Asia (n=5) | 1.29 (0.64-2.59) | 0.481 | No | --- | | |
| Europe (n=4) | 1.12 (1.02-1.22) | 0.013 | Yes | Sorahan et al., 2005 [37] | 1.10 (1.00-1.21) | 0.058 |
| **Mortality** (n=13) | 1.15 (1.02-1.30) | 0.023 | Yes | Sorahan et al., 2005 [37] | 1.13 (0.98-1.31) | 0.093 |
| **Smoking** | | | | | | |
| Adjusted (n=1) | | | --- | | | |
| Not adjusted (n=12) | 1.17 (1.01-1.35) | 0.037 | Yes | Sorahan et al., 2005 [37] | 1.14 (0.94-1.37) | 0.178 |
| **Exposure** | | | | | | |
| Residential (n=1) | | | --- | | | |
| Occupational (n=12) | 1.17 (1.01-1.35) | 0.037 | Yes | Sorahan et al., 2005 [37] | 1.14 (0.94-1.37) | 0.178 |
| Others: biomarkers (n=0) | | | | | | |
| **Sex** | | | | | | |
| Men (n=7) | 1.03 (0.77-1.37) | 0.867 | No | --- | | |
| Women (n=2) | 1.38 (0.87-2.17) | 0.167 | --- | | | |
| Men and Women (n=4) | 1.14 (1.00-1.29) | 0.049 | Yes | Sorahan et al., 2005 [37] | 1.11 (0.94-1.32) | 0.229 |
| **Study design** | | | | | | |
| Case Control (n=1) | | | --- | | | |
| Cohort (n=12) | 1.17 (1.05-1.32) | 0.006 | No | --- | | |
| **Region** | | | | | | |
| North America (n=6) | 1.07 (0.93-1.24) | 0.343 | No | --- | | |
| Asia (n=6) | 1.21 (0.91-1.61) | 0.199 | Yes | Koh et al., 2011 [35] | 1.38 (1.10-1.71) | 0.004 |
| Europe (n=1) | | | | | | |
